# Species-specific acoustic responses by bats to ultrasonic stimuli used for reducing bat-wind turbine interactions

**DOI:** 10.7717/peerj.21231

**Published:** 2026-04-28

**Authors:** Emma E. Guest, Sara P. Weaver, Amanda Marie Hale, Brogan Page Morton, Cris Daniel Hein, Sarah Rebecah Fritts

**Affiliations:** 1 Bowman Consulting, San Marcos, TX, United States of America; 2 Energy Renewal Partners, Austin, TX, United States of America; 3 Department of Biology, Texas State University, San Marcos, TX, United States of America; 4 Western Ecosystems Technology, Inc., Cheyenne, WY, United States of America; 5 Department of Biology, Texas Christian University, Fort Worth, TX, United States of America; 6 Wildlife Imaging Systems, Hinesburg, VT, United States of America; 7 National Renewable Energy Laboratory, Golden, CO, United States of America

**Keywords:** Bats, Wind energy, Chiroptera, Ultrasonic deterrents, Climate change, Renewable energy, Turbine blade

## Abstract

An unintended consequence of wind energy generation is bat fatalities caused by wind turbine blade strikes. One potential approach to reduce collision risk is to use ultrasound to create an uncomfortable or disorienting airspace around wind turbine blades. Ultrasonic deterrents (UDs) have produced mixed results in experimental field studies at commercial wind energy facilities, with effectiveness varying by species and location. It is possible that some species can alter their normal echolocation characteristics to counter the signal of UDs. Our broad objective was to maximize the effectiveness of a UD by comparing changes in echolocation characteristics during three UD frequency emissions among species, between seasons, and between sex. We hypothesized that UD emissions with frequencies most similar to each species’ echolocation characteristics would be more likely to alter the bats’ echolocation, and bat responses would vary between seasons and sex for each species. We released wild-captured bats into a 60 m × 10 m × 4.4 m (length × width × height) flight cage located in San Marcos, Texas, USA, from July to October 2020 and March to May 2021 and monitored echolocation frequencies with ultrasonic microphones. We conducted trials on Brazilian free-tailed bats (*Tadarida brasiliensis*; *n* = 54), cave myotis (*Myotis velifer*; *n* = 44), red bats (*Lasiurus blossevilli, Lasiurus borealis*; *n* = 41), evening bats (*Nycticeius humeralis*; *n* = 32), and tricolored bats (*Perimyotis subflavus*; *n* = 8). We found that species with high-frequency echolocation calls altered their echolocation signatures more commonly during high-frequency UD emissions, whereas low-frequency bats altered their echolocation signatures more commonly during low-frequency UD emissions. Additionally, echolocation responses varied between seasons and sexes for several species. Variations in responses may be dependent on species migratory status, differences in mating behavior and mating season, hormonal differences between sexes and seasons, and/or constraints on echolocation adaptability. Our results offer insights into the variable effectiveness of UDs at reducing bat fatalities at wind turbines and provide information for potential adjustments to UDs for improved success.

## Introduction

Wind energy production generates zero emissions during operation and has competitive energy pricing; thus, global deployment is expanding (International Energy Agency, 2020). Although renewable energy is important for energy diversification and to meet growing global demand for electricity, wind energy is not without unintended negative impacts on wildlife (Allison et al., 2019). One consequence of wind energy operation is bat fatalities caused by wind turbine blade strikes. Increasing wind energy development represents a stressor to numerous bat species, sparking concern among conservationists and the wind energy industry (Frick et al., 2017). In the US and Canada, migratory tree-roosting species, including the hoary bat (*Lasiurus cinereus*), eastern red bat (*Lasiurus borealis*), and silver-haired bat (*Lasionycteris noctivagans*), constitute most of the bat carcasses reported in the US and Canada from wind turbine strikes and are therefore thought to be the most vulnerable species to wind turbine-related fatality in these countries (Arnett et al., 2005; Zimmerling & Francis, 2016; American Wind Wildlife Institute, 2020; Choi, Wittig & Kluever, 2020; Endangered Species Recovery Committee and State of Hawaii Department of Land and Natural Resources Division of Forestry and Wildlife, 2021). Additionally, wind turbine strikes are known to cause fatality of protected species, including the Hawaiian hoary bat (*Lasiurus semotus*), northern long-eared bat (*Myotis septentrionalis*), and Indiana bat (*M. sodalis*) (Erickson et al., 2016; American Wind Wildlife Institute, 2020; Gorresen, Cryan & Tredinnick, 2020). Bat fatality due to wind turbines has been reported across all continents (Arnett et al., 2016), excluding Antarctica, highlighting the potential global effect of wind turbines on bat populations.

Bats use high-frequency sound, or echolocation, for navigation, locating food and water, and a variety of social interactions (Schnitzler & Kalko, 2001; Schnitzler, Moss & Denzinger, 2003). Bats emit calls and analyze the returning echo to visualize the surrounding environment (Schnitzler, Moss & Denzinger, 2003). The range of frequencies used and the structure of calls that bats produce vary among species (Schnitzler & Kalko, 2001). Echolocation behavior of a particular species primarily depends on feeding behavior and habitat type (Schnitzler & Kalko, 2001; Schnitzler, Moss & Denzinger, 2003). The species-specific nature of echolocation still allows for intraspecific variation to exist. A single species, as well as a single individual within a species, can produce calls of varying frequency, length, and sound pressure level depending on the type of call, location, and task (Jones, Vaughan & Parsons, 2000; Schnitzler & Kalko, 2001; Schnitzler, Moss & Denzinger, 2003). Researchers can access this information for species and activity assessments using acoustic detectors (Jones, Vaughan & Parsons, 2000). Such detectors record ultrasonic activity; when these detectors are used in conjunction with acoustic analysis software that creates and visualizes bat call files, bat calls can often be identified to species and assigned to an activity category (*e.g.*, commuting, foraging) based on a range of characteristics (Jones, Vaughan & Parsons, 2000; Adams, 2013).

NRG Systems (NRG) developed an ultrasonic deterrent (UD) for installation on wind turbines in an attempt to reduce bat fatalities from wind turbine blade strikes. The current UD design consists of six ultrasonic speakers, or “subarrays”, each capable of emitting a constant frequency of 20, 26, 32, 38, 44, or 50 kHz, thus cumulatively emitting a range of 20 to 50 kHz, encompassing the characteristic frequencies of most North American bat species (California State Polytechnic University & Humboldt State Bat Lab, 2024; California State Polytechnic University & Humboldt State Bat Lab, 2011). The NRG deterrent produces ultrasonic sound at a sound pressure level of 120 decibels (dB) at a 1-m distance, with intensity attenuating at a rate dependent on weather variables and frequency. Previous research suggests UDs may lower overall bat fatalities at wind energy facilities, but species-specific effectiveness is variable (Romano et al., 2019; Weaver et al., 2020; Clerc et al., 2025). Reasons for species-specific variability are not well understood, but it could be due to certain species’ abilities to alter echolocation characteristics in response to UDs (Gilmour et al., 2021). Additionally, high-frequency sound waves attenuate more rapidly as frequency increases and potentially do not reach past or extend to the perimeter of the rotor-swept zone of most wind turbine blades (Weaver et al., 2020). This could mean that only the lower-frequency range of sounds emitted from the UD is effective at deterring low-frequency bat species. An analysis of echolocation characteristics from bats exposed to ultrasonic stimuli may reveal key insights for developing a more effective form of this technology to reduce wind turbine-related fatalities.

As part of a larger study to assess species-specific effectiveness of the NRG UD on bats, our objective was to understand the role that echolocation response could play as a potential mechanism for UD success. The larger study examined paired flight and echolocation behavior in bats exposed to various UD emissions in a flight cage experiment. For the echolocation portion of the study that we report herein, our research questions were: (1) Do bats alter their echolocation frequency based on UD frequency emission? (2) Do echolocation alterations differ by season and/or sex? and (3) How do these echolocation results compare to observations of flight behavior from Fritts et al. (2024)? We hypothesized that bats would alter their echolocation frequency during UD emission and predicted that high-frequency treatments would have a greater influence on species that echolocate at high frequencies, whereas low-frequency treatments would have a greater influence on species that echolocate at low frequencies. Additionally, we predicted that both high- and low-frequency species would lower their echolocation frequency to resist UD interference, because low-frequency sounds allow for increased range for orientation (Griffin, 1971). However, we expected bats would be more affected by the frequency emission (*i.e.,* treatment) that is nearer to the characteristic frequency of their echolocation calls. We assessed the difference between sex and season to determine whether one of the treatments would be more effective for female bats during the fall testing season. Given most bat fatalities from wind turbine blade strikes occurs during the late summer through early fall (Arnett et al., 2008; Arnett et al., 2013) and bats have a low reproductive rate (Barclay & Harder, 2003), it is important to minimize impacts to female bats. Portions of this text were previously published as part of a thesis (Guest, 2021).

## Materials & Methods

**Figure 1:**
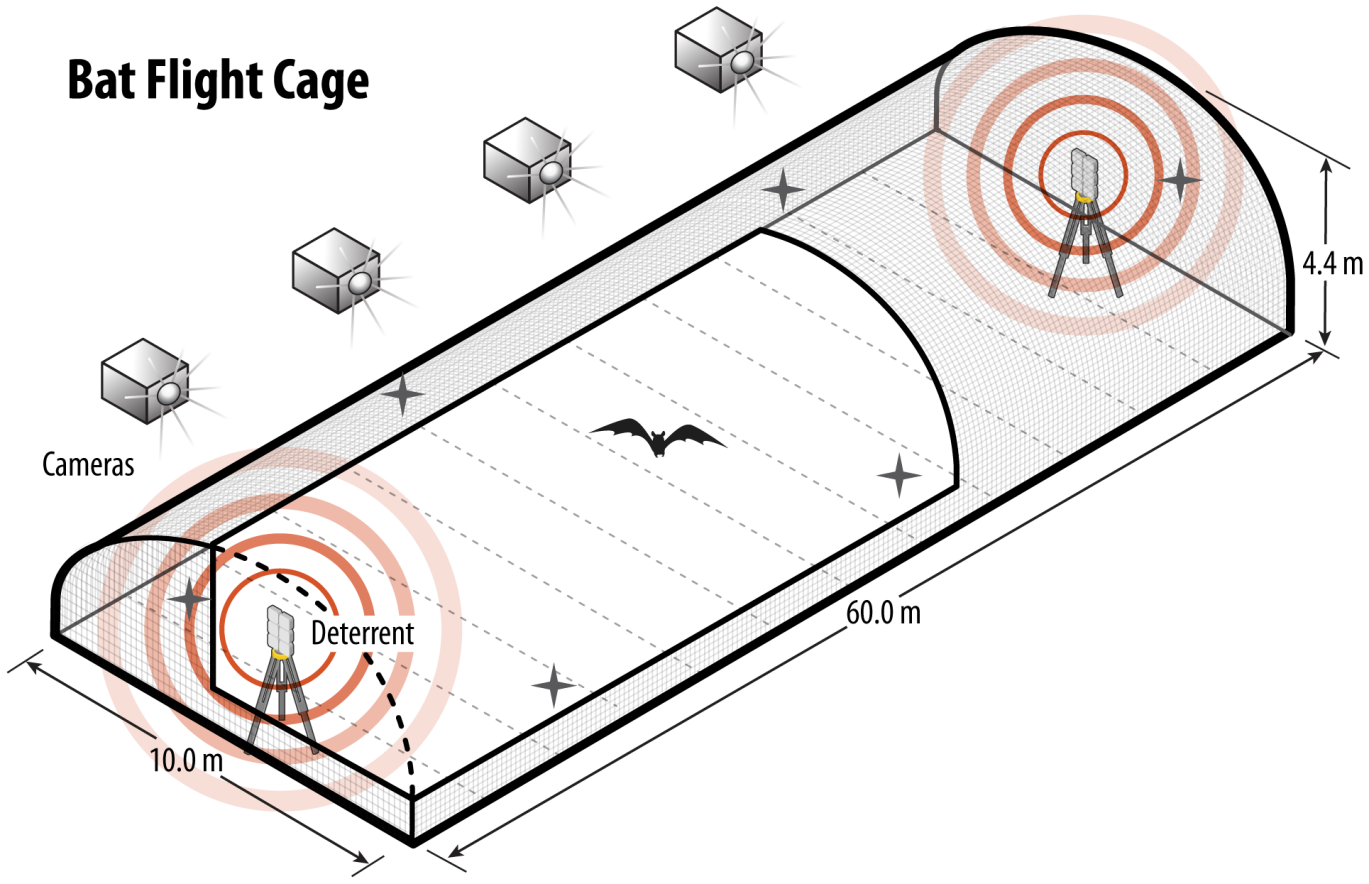
Schematic of the flight cage used to assess flight and echolocation responses of bats to ultrasonic deterrent emissions. Schematic of flight cage to test bat echolocation responses to an ultrasonic deterrent. The flight cage was (*l* × *w* × *h*) 60 × 10 × 4 m with four thermal cameras deployed outside of the cage to track bat flight and three, Wildlife Acoustic SM3 bat detectors, each with two microphones (denoted by the crosses), and an ultrasonic deterrent (UD) on either side of the cage. We selected one UD for each night of trials.

We collected data over two consecutive field seasons, July–October 2020 and March–May 2021, hereafter referred to as “fall” and “spring”, respectively. The team constructed a flight cage in an open grass field located at the Freeman Center, a 1,416-hectare ranch owned and operated by Texas State University (see Fritts et al., 2024). The flight cage was 60 m × 10 m × 4.4 m (length × width × height). We based the cage dimensions on the length of a relatively long land-based wind turbine blade to ensure the UD emission would reach the blade tips and thus cover the entire rotor swept area. However, as land-based wind turbines have rapidly increased in size, many now have blades that are greater than 60 m in length and thus the extent of the flight cage does not capture the maximum blade length that bats could encounter on the landscape. The width of the flight cage was as wide as possible without requiring center support structures, which would have potentially interfered with flight paths and sound transmission from the deterrent to the test subjects. Additionally, the width allowed bats to turn around easily within the flight cage. We constructed the cage using aluminum posts covered with 0.635-cm lightweight plastic mesh. We positioned a UD at each end of the flight cage. The UDs were mounted on two T-posts approximately 1.5 m off the ground (Fig. 1). We ensured the ground was level and removed vegetation throughout the duration of the study to promote normal bat behavior inside the flight cage.

We positioned three Wildlife Acoustics (Maynard, Massachusetts, USA) Song Meter SM3BAT detectors, each equipped with two SMM-U2 Wildlife Acoustics ultrasonic microphones, every 15 m along the inside perimeter of the long axis of the flight cage approximately 1.8 m above the ground, for a total of six microphones. We positioned two of the six microphones at each end just behind each UD, adhered to painters’ poles and suspended approximately 3.5 m above the ground. We calibrated each microphone using the Wildlife Acoustics ultrasonic calibrator on “calibrate” mode prior to installation inside the flight cage. Additionally, we used the calibrator to test for potential acoustic detector “blind spots” prior to data collection. We set the calibrator to chirp mode and switched it on while we stood at various locations within the flight cage and recorded the time and location of each chirp both with and without the UD turned on. We analyzed the chirp files using SonoBat (SonoBat 4.2.2.1 North America, Arcata, CA, USA) analysis software to ensure the microphone positions would record calls across the entirety of the flight cage. The calibrator set on chirp mode is unidirectional and emits a 40 kHz signal at a sound pressure level of 100 dB. We used a GPS time-sync receiver prior to each night of trials. The GPS receiver synchronized each SM3BAT detector to the GPS time base to allow for accurate synchronization across all detectors. Time synchronization was important because a single bat call can be recorded by multiple microphones in proximity, so synchronized time stamps on recorded files allowed duplicate files to be accounted for. We performed research under state (Texas State SPR-1217-243) and IACUC (Texas State IACUC protocol 6224) permits for handling bats and adhered to decontamination guidelines in the United States National White-nose Syndrome Decontamination Protocol (White-Nose Syndrome Response Team, https://www.whitenosesyndrome.org/).

We captured bats using a variety of methods, including mist nets, harp traps, hand nets, and extraction from culverts, bridges, caves, and across various habitats where bats were active. Capture locations were within a 2-hour drive from the Freeman Center in south-central Texas. After capture, we placed bats in cloth bags and transported them to the flight cage where we recorded species, age, sex, reproductive condition, and right forearm length and noted any abnormalities such as wing damage or significant scarring for each captured individual prior to the start of the UD trials. Due to capture success, we focused analyses on five species: Brazilian free-tailed bats (*Tadarida brasiliensis*), cave myotis (*Myotis velifer*), red bats (*Lasiurus* spp.), evening bats (*Nycticeius humeralis*), and tricolored bats (*Perimyotis subflavus*). Since this study was initiated, it was discovered that our capture locations are in a region of sympatry for eastern red bats and western red bats (*L. blossevillii*; Guest et al., 2025). While eastern red bats have been widely documented across Texas, prior to Guest et al. (2025) the western red bat had only been recorded in western and far southern Texas. These two species can be difficult to distinguish based on physical characteristics. Because we did not confirm species identification for all captured red bats, we analyzed eastern and western red bats as one species group (*i.e.,* red bats), as was done in Fritts et al. (2024). Our focal species represent various echolocation call frequencies with Brazilian free-tailed bats having characteristic and high frequencies of approximately 25 and 31 kHz, cave myotis of 42 and 76 kHz, red bats of 43 and 65 kHz, evening bats of 38 and 63 kHz, and tricolored bats of 43 and 58 kHz (California State Polytechnic University & Humboldt State Bat Lab, 2024; California State Polytechnic University & Humboldt State Bat Lab, 2011).

For the UD trials, we randomly selected one of the two deterrents located at either end of the flight cage to be used for the trials each night. We released a single captured individual in the flight cage for a total of 32 min from time of entry to time of last control period. We began the trial with a 4-min acclimation period to give individual bats an opportunity to adjust to the flight cage. The acclimation period was followed by a 4-min control period to establish a baseline of echolocation data to be compared to the data from treatment periods. Each trial consisted of three randomly selected continuous treatments: (1) 20–50 kHz (combined), (2) 20–32 kHz (low), and (3) 38–50 kHz (high). Each treatment lasted 4 min and was followed by a 4-min recovery period with no UD emission. The three treatments were chosen to determine if low-frequency treatments would be effective for altering the behavior of high-frequency bat species; the low-frequency treatment is preferred because low-frequency sound travels farther due to less attenuation (Hartley, 1989). Data from individuals that quit flying or roosted on the netting of the flight cage were omitted from the study.

Following each trial, we used hand nets to safely capture the individual so that it could be removed from the flight cage. After the UD trials ended, we offered the bats water and meal worms (*Tenebrio molitor*) and then returned them to their capture site for release that same night.

The full-spectrum acoustic recordings collected during the UD trials were input into Wildlife Acoustics Kaleidoscope Pro (version 5.4.1) acoustic analysis software; however, while bat acoustic software is convenient for extracting non-bat “noise”, we also manually vetted each recording to visually assess the software results and ensure we were not including calls from bats outside of the flight cage. This software program can split files based on the microphone that recorded each bat call. Bat calls from outside the flight cage were primarily from Brazilian free-tailed bats, and because we knew the species identification of the bat inside the flight cage during each trial, we were able to differentiate between calls from differing species. We were able to discern calls from the bat in trial from calls of outside bats of the same species, as outside bat calls were farther away from the microphones and therefore had lower sound pressure level. To separate quantitative data concerning the individual of interest from free-flying bats that were echolocating outside the flight cage, we analyzed acoustic files by pulse as opposed to call sequence. To do this, we processed the manually vetted acoustic files using the SonoBatch function of SonoBat acoustic analysis software and employed an output from the software that provides quantitative characteristics for each pulse, as opposed to quantitative characteristics from the overall call file. From there, we were able to use the file label we previously created during the manual vetting process, in combination with quantitative metrics of each pulse, to remove output that belonged to outside bats. Additionally, we removed all output rows that contained a bandwidth value of <1 kHz, as this value corresponded to the known constant-frequency noise produced by the UD (verified during UD-only recordings) that the software had incorrectly identified as a bat pulse. Lastly, we used number of pulses in a particular file as a proxy for call file quality, which allowed us to select the top-quality file and remove any duplicate files.

We used R (https://www.r-project.org/, version 1.4.1717) for statistical computation and conducted models for each species and species group with five echolocation characteristics as our response variables: minimum frequency (Fmin), maximum frequency (Fmax), characteristic frequency (Fc), difference between Fmax and Fmin (Fdiff), and pulse duration (PD). Minimum frequency is the lowest apparent frequency of a given pulse, and maximum frequency is the greatest frequency of a given pulse. Characteristic frequency is the frequency at the end of the body of a pulse and is an important call characteristic for manual identification. Lastly, pulse duration is the duration of a given pulse recorded in milliseconds. We used the mean of each response for each individual to prevent pseudoreplication. First, we checked for normality of response variables using a Shapiro–Wilks test and homogeneity of variances across groups using a Levene’s test. Several responses failed the Shapiro–Wilks test and were then assessed visually using a QQplot. No responses were drastically different from normal, and because Analysis of Variance is robust to slight variations from normality, we proceeded with ANOVAs with interactive effects among treatment and sex and treatment and season for each response variable for each species. For red bats, we examined the potential interactive effects between sex and season because that interaction was significant in the flight behavior model in Fritts et al. (2024). For each species and species group, we then ran a model with only significant variables and compared means using Tukey’s test, which corrects for experimental-wise error rates. We used a QQplot to assess normality of residuals to ensure assumptions were met. We further visualized results using bar plots with pairwise significant tests and a Bonferonni correction. Additionally, we used a Benjamini–Hochberg false discovery rate adjustment for the *p*-values. We also report the number of pulses recorded by each of the detectors and whether the detector was near the deterrent (near), in the middle of the flight cage (middle), or on the opposite side of the flight cage (far).

## Results

**Figure 2:**
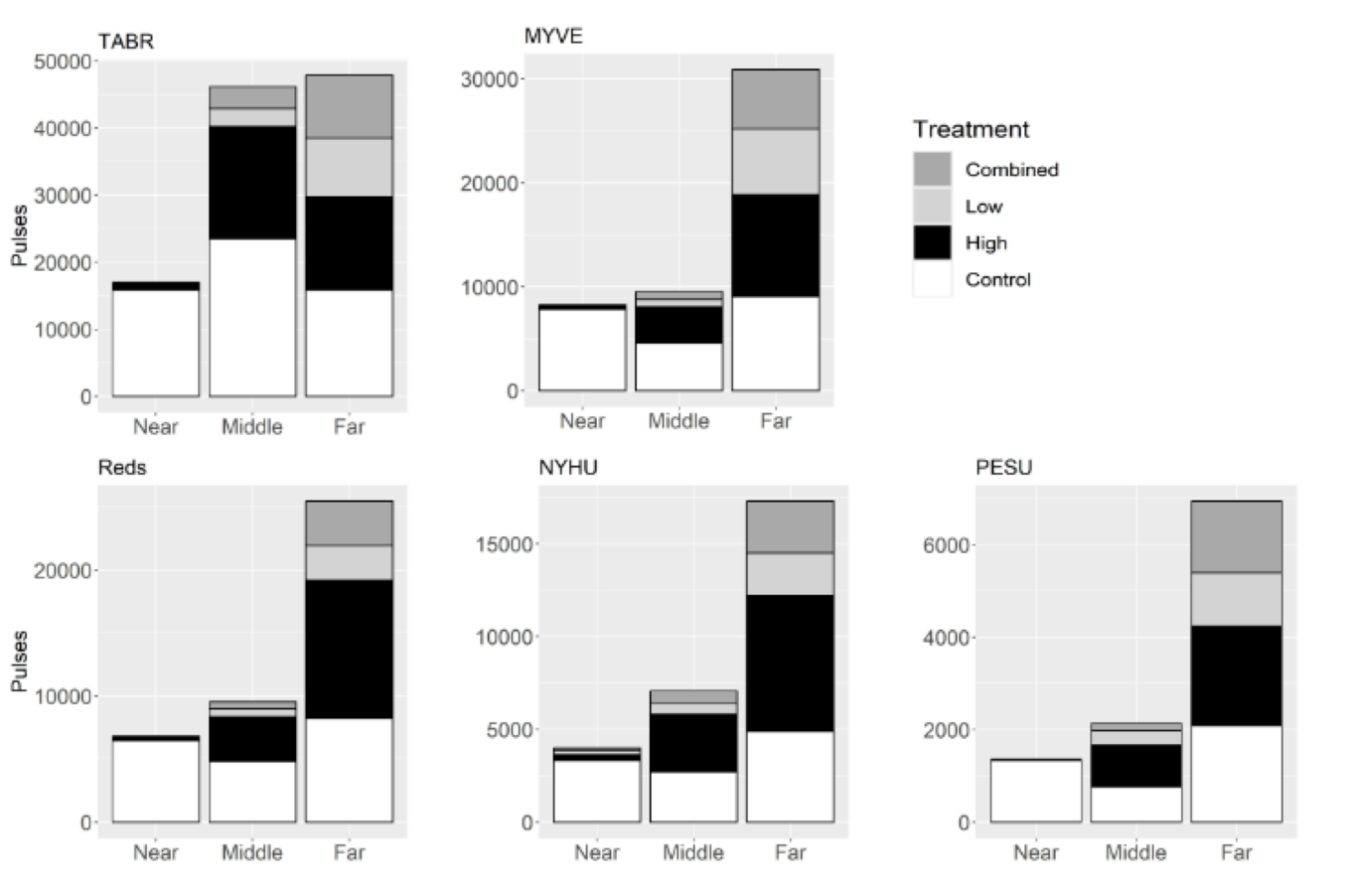
Number of echolocation pulses by species from each of three acoustic detectors during each ultrasonic deterrent treatment. Number of pulses recorded on each acoustic detector inside the flight cage by species and ultrasonic deterrent (UD) treatment. The three acoustic detectors each had two microphones and were placed on either the side with the UD (“near”), in the middle of the flight cage (“middle”), or the side without the UD (“far”). TABR = *Tadarida brasiliensis*; MYVE = *Myotis velifer*; red bats = *Lasiurus borealis* and *L. blossevillii*); NYHU = *Nycticeius humeralis*; PESU = *Perimyotis subflavus*. Treatments include Control and ultrasonic deterrent emissions (Low (20–32 kHz), High (38–50 kHz), and Combined (20–50 kHz)).

After omitting data from bats that did not fly continuously during the UD trials, we analyzed data for Brazilian free-tailed bats (*n* = 54 after four omitted), cave myotis (*n* = 44 after 10 omitted), red bats (*n* = 41 after three omitted), evening bats (*n* = 32 after seven omitted), and tricolored bats (*n* = 8 after zero omitted) (Table 1). Results are summarized in Table 2 and the number of pulses during each of the treatments for each species are displayed in Fig. 2. All species had the greatest number of pulses detected during the Control treatment followed by the High treatment. The far microphone detected the greatest number of pulses for all species.

**Table 1:** Sample size by sex and season for each species and species group included in the study.

Species	Male		Female	
	Fall 2020	Spring 2021	Fall 2020	Spring 2021
Brazilian free-tailed bat	19	4	18	13
Cave myotis	18	8	18	0
Red bat	14	4	12	11
Evening bat	14	0	13	5
Tricolored bat	4	1	2	1

**Table 2:** ANOVA results that compared echolocation characteristics of bats during ultrasonic acoustic trials. Species included Brazilian free-tailed bats (*Tadarida brasiliensis*; TABR), cave myotis (*Myotis velifer*; MYVE), red bats (*Lasiurus borealis*/*Lasiurus blossevillii*; Reds), and evening bats (*Nycticeius humeralis*; NYHU). Treatments included three ultrasonic emissions from the NRG Systems ultrasonic deterrent (L ow: 20, 26, and 32 kHz; High: 38, 44, and 50 kHz; Combined: 20, 26, 32, 38, 44, and 50 kHz) to a control period of no emissions. We included include main effects for treatment (trt) and interactive effects for trt:sex and trt:season (fall *vs* spring). The estimated effects are averages across individual bats for the different quantiles of control minus treatment differences in distance. Trials were conducted in a flight cage from July 2020 to May 2021 in San Marcos, Texas (USA).

		TABR		MYVE		NYHU		Reds	
Response	Independent variable	F-statistic	*p*-value	F-statistic	*p*-value	F-statistic	*p*-value	F-statistic	*p*-value
Fc	Trt	145.250	**<0**.**001**	4.425	**<0**.**025**	6.512	**<0**.**001**	0.705	0.868
Fc	Sex	15.490	**<0**.**001**	6.418	**<0**.**045**	18.584	**<0**.**001**	116.740	**<0**.**001**
Fc	Season	3.640	0.420	0.052	0.909	7.597	0.032	2.342	0.320
Fc	Trt:Sex	0.980	0.909	0.290	0.909	0.059	0.989	0.140	0.964
Fc	Trt:Season	1.880	0.766	0.359	0.909	0.285	0.909	0.274	0.909
Fc	Sex:Season							2.412	0.315
Fmax	Trt	16.947	**<0**.**001**	3.837	**<0**.**043**	1.174	0.644	5.234	**<0**.**012**
Fmax	Sex	3.035	0.255	3.115	0.251	2.783	0.264	27.662	**<0**.**001**
Fmax	Season	0.411	0.868	9.078	0.016	0.367	0.868	0.975	0.644
Fmax	Trt:Sex	0.317	0.909	0.356	0.909	0.467	0.909	0.119	0.967
Fmax	Trt:Season	0.456	0.909	0.387	0.909	0.510	0.909	0.589	0.896
Fmax	Sex:Season							0.249	0.896
Fmin	Trt	125.352	**<0**.**001**	1.452	0.525	13.331	**<0**.**001**	0.965	0.766
Fmin	Sex	6.852	**<0**.**042**	5.306	**<0**.**078**	11.375	**<0**.**006**	143.185	**<0**.**001**
Fmin	Season	1.017	0.644	2.838	0.260	11.162	**<0**.**006**	7.570	0.032
Fmin	Trt:Sex	1.360	0.572	0.041	0.989	0.343	0.909	0.181	0.945
Fmin	Trt:Season	1.746	0.388	0.257	0.909	0.642	0.873	0.272	0.909
Fmin	Sex:Season							3.013	0.255
Fdiff	Trt	1.634	0.427	3.802	0.043	0.429	0.777	145.950	0.016
Fdiff	Sex	0.226	0.901	6.020	0.054	0.751	0.909	89.370	0.255
Fdiff	Season	1.177	0.598	7.081	**<0**.**039**	0.090	0.255	100.100	0.236
Fdiff	Trt:Sex	0.519	0.909	0.326	0.909	0.578	0.868	6.550	0.930
Fdiff	Trt:Season	1.089	0.690	0.404	0.909	0.530	0.868	22.940	0.868
Fdiff	Sex:Season							31.660	0.644
PD	Trt	17.691	**<0**.**001**	3.404	0.066	20.205	**<0**.**001**	12.563	**<0**.**001**
PD	Sex	0.037	0.909	13.045	**<0**.**001**	0.309	0.868	2.642	0.278
PD	Season	0.327	0.868	0.115	0.909	0.755	0.739	12.850	**<0**.**001**
PD	Trt:Sex	0.665	0.868	0.745	0.868	0.551	0.909	0.380	0.909
PD	Trt:Season	0.461	0.909	1.303	0.598	0.437	0.909	0.793	0.868
PD	Sex:Season							0.388	0.868

**Notes.**

Significant differences are indicated in bold.

**Figure 3:**
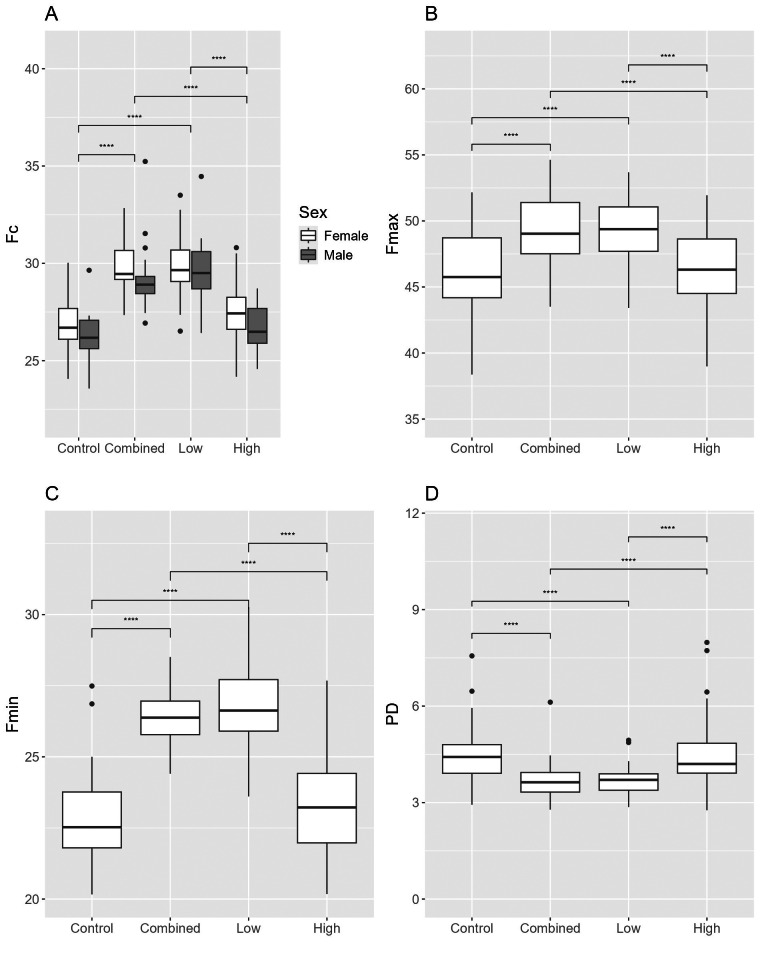
Differences in echolocation characteristics of Brazilian free-tailed bats (*Tadaria brasilienses*) during ultrasonic deterrent trials. Asterisks indicate significant differences (* = 0.05, ** = 0.01, *** = 0.001, and **** = 0.0001). Treatments included Control and ultrasonic deterrent emissions (Low (20–32 kHz), High (38–50 kHz), and Combined (20–50 kHz)). (A) characteristic frequency (Fc), (B) maximum frequency (Fmax), (C) minimum frequency (Fmin), and (D) pulse duration (PD). Significant differences are indicated above the box plots.

For Brazilian free-tailed bats, there were 3,217 recordings that included 110,952 pulses (Control = 55,096; Combined = 12,611; Low = 11,520; High = 31,725). The Control treatment had relatively equal numbers of pulses recorded by the near and far microphones with more pulses in the middle; the High treatment had a greater number of pulses detected by both the middle and far microphones; and the Low and Combined treatments had a greater number of pulses detected by the far microphone (Fig. 2). Fmin, Fmax, and Fc were higher during treatments that were more similar to their echolocation call characteristics: Low and Combined treatments compared to Control and High treatments (Table 2; Figs. 3A–3D). PD was longer during Control and High treatments than during Low and Combined treatments. Fc was higher in females, and both treatment and sex influenced Fc and Fmin with no interactive effects (Figs. 3A and 3C). Fmax results were similar, except sex was not significant (Fig. 3B). There were no interactive effects for PD (Fig. 3D), and Fdiff was not influenced by treatment, sex, or season.

**Figure 4:**
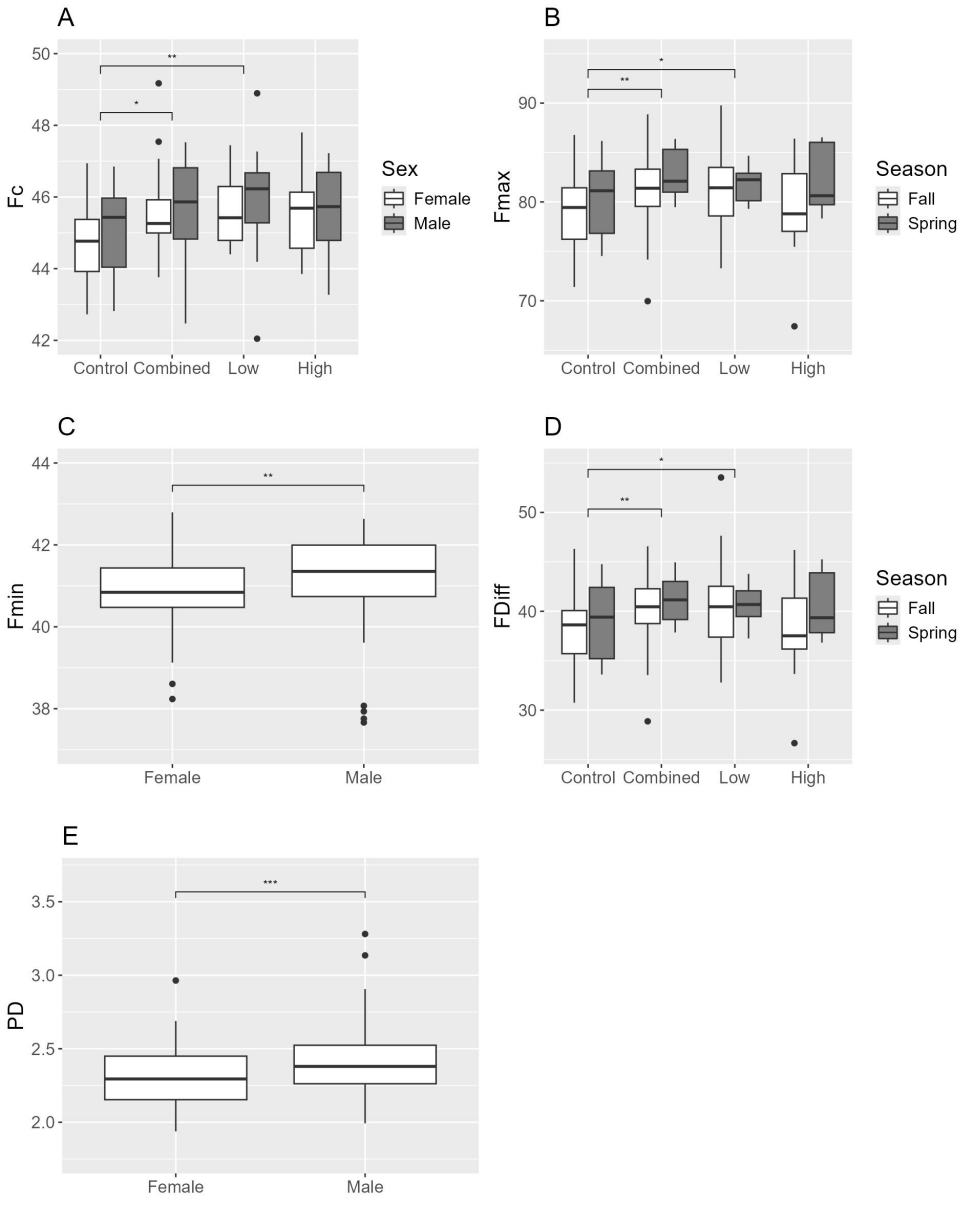
Differences in echolocation characteristics of cave myotis (*Myotis velifer*) during ultrasonic deterrent trials. Asterisks indicate significant differences (* = 0.05, ** = 0.01, *** = 0.001, and **** = 0.0001). Treatments included Control and ultrasonic deterrent emissions (Low (20–32 kHz), High (38–50 kHz), and Combined (20–50 kHz)). (A) characteristic frequency (Fc), (B) maximum frequency (Fmax), (C) minimum frequency (Fmin), (D) difference between Fmax and Fmin (Fdiff), and (E) pulse duration (PD). Significant differences are indicated above the box plots.

For cave myotis, there were 1,602 recordings that contained 48,695 pulses (Control = 21,382; Combined = 6,495; Low = 7,047; High = 13,771). The Control treatment had relatively equal numbers of pulses detected by the near and far microphones with fewer pulses in the middle; the High treatment had greater numbers of pulses detected by both the middle and far microphones than the near; and the High, Low and Combined treatments had the greatest number of pulses detected by the far microphone (Fig. 2). Fc, Fmax, and Fdiff differed between treatments and Control; Fc differed among all treatments and Control, whereas Fmax and Fdiff differed between Combined and Low treatments and Control (Table 2; Figs. 4A–4E). PD differed between sexes (Fig. 4E). In fall, females had greater Fc than males, whereas males had longer PD and Fmin than females (Figs. 4A and 4E). For males, Fmax was greater in spring than fall (Fig. 4B). We did not capture females during spring and thus did not assess the interactive effect of sex and season.

**Figure 5:**
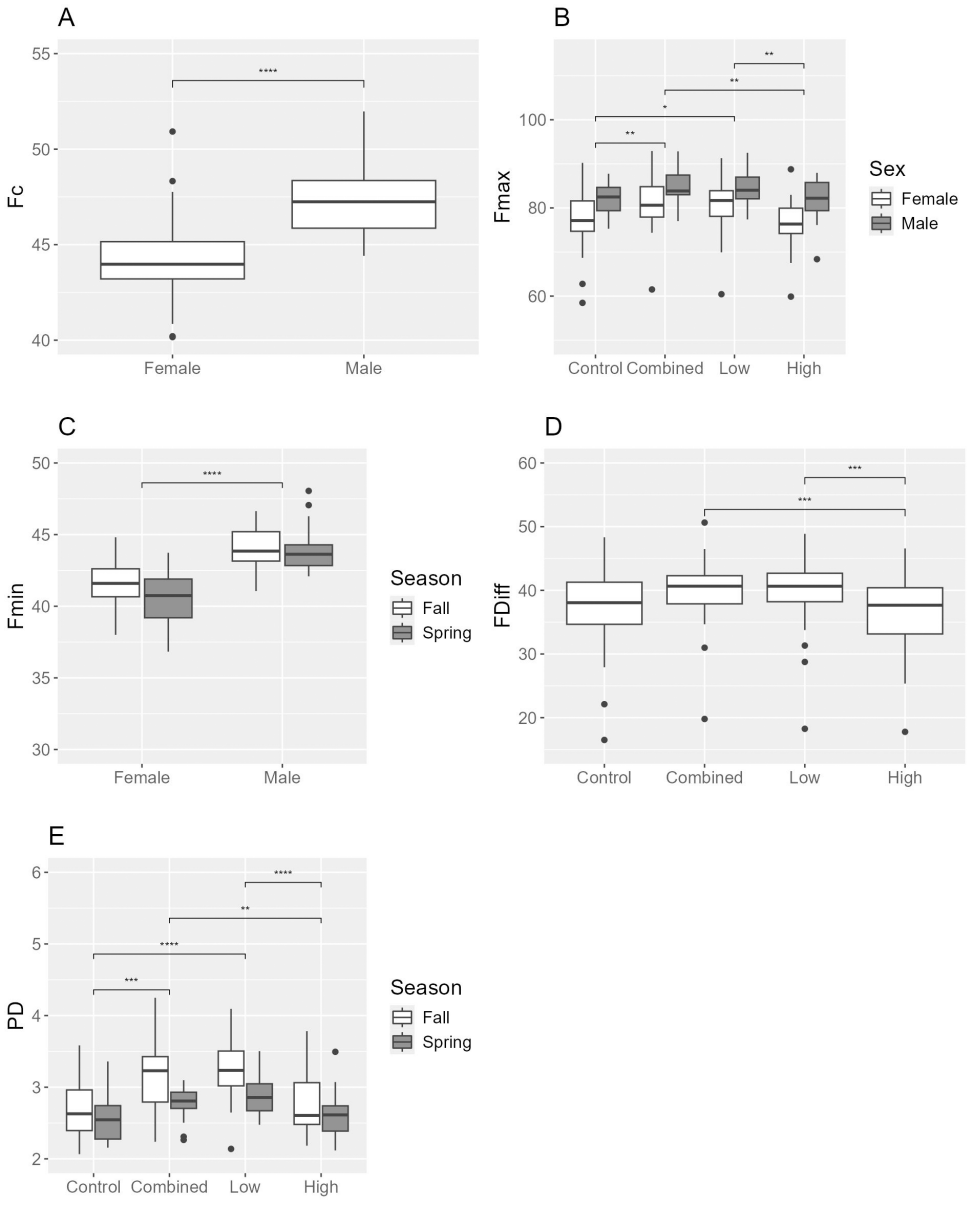
Differences in echolocation characteristics of red bats (*Lasiurus borealis* and *L. blossevillii*) during ultrasonic deterrent trials. Asterisks indicate significant differences (* = 0.05, ** = 0.01, *** = 0.001, and **** = 0.0001). Treatments included Control and ultrasonic deterrent emissions (Low (20–32 kHz), High (38–50 kHz), and Combined (20–50 kHz)). (A) characteristic frequency (Fc), (B) maximum frequency (Fmax), (C) minimum frequency (Fmin), (D) difference between Fmax and Fmin (Fdiff), and (E) pulse duration (PD). Significant differences are indicated above the box plots.

For red bats, there were 2,127 recordings containing 41,813 pulses (Control = 19,517; Combined = 4,063; Low = 3,446; High = 14,787). The Control treatment had relatively equal numbers of pulses detected by the near and far microphones with fewer pulses in the middle; the High treatment had greater numbers of pulses detected by both the middle and far microphones than the near; and the High, Low and Combined treatments had the greatest number of pulses detected by the far microphone (Fig. 2). Responses were greater in treatments that were not similar to their echolocation frequencies with Fmax being higher in Combined and Low treatments *versus* Control, and PD being longer in all treatments compared to Control (Table 2; Figs. 5A–5E). No other response varied between treatments and Control. Fmax and Fdiff were lower and PD was shorter in High *versus* Low treatments (Figs. 5B, 5D, and 5E). Fc and Fmax were higher in males than females (Figs. 5A and 5B). Fmin and PD were greater in spring *versus* fall (Figs. 5C and 5E). There were no interactive effects in any response between sex and season.

**Figure 6:**
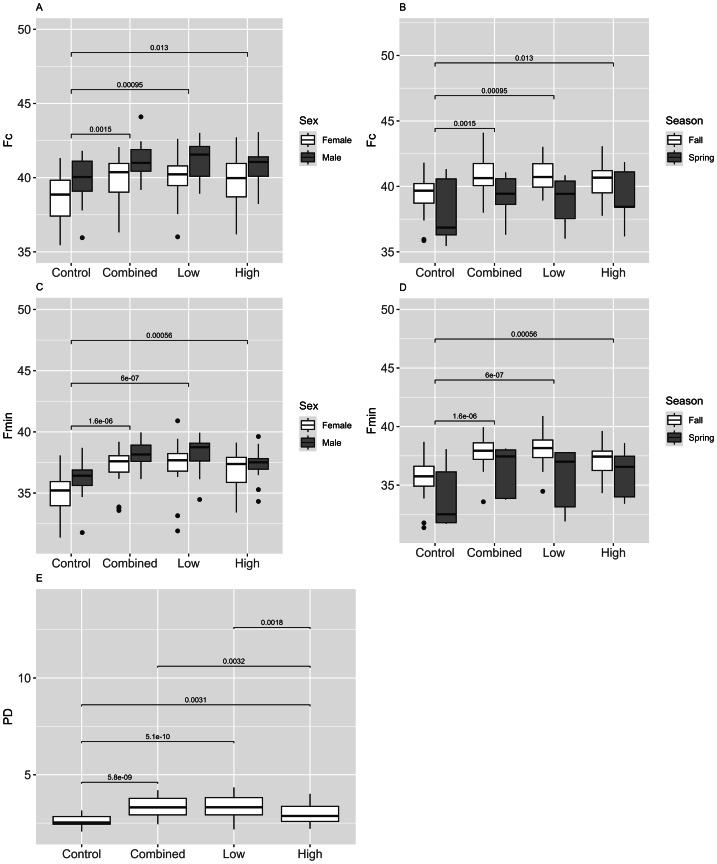
Differences in echolocation characteristics of evening bats (*Nycticeius humeralis*) during ultrasonic deterrent trials. Asterisks indicate significant differences (* = 0.05, ** = 0.01, *** = 0.001, and **** = 0.0001). Treatments included Control and ultrasonic deterrent emissions (Low (20–32 kHz), High (38–50 kHz), and Combined (20–50 kHz)). (A) characteristic frequency (Fc), (B) maximum frequency (Fmax), (C) minimum frequency (Fmin), (D) difference between Fmax and Fmin (Fdiff), and (E) pulse duration (PD). Significant differences are indicated above the box plots.

For evening bats, there were 1,800 recordings containing 28,349 pulses (Control = 10,929; Combined = 3,576; Low = 3,123; High = 10,721). The Control treatment had relatively equal numbers of pulses detected by the near and far microphones with fewer pulses in the middle; the High treatment had greater numbers of pulses detected by both the middle and far microphones than the near; and the High, Low and Combined treatments had the greatest number of pulses detected by the far microphone (Fig. 2). Fc and Fmin were and PD was longer in all treatments *versus* Control (Figs. 6A–6C). Fc and Fmin were higher in males than females (Table 2; Figs. 6A1 and 6B1) and during fall compared to spring (Figs. 6A2 and 6B2). We did not include the interaction between sex and season in the ANOVA because of the low sample size of spring males.

For tricolored bats, there were 472 recordings containing 10,431 pulses (Control = 4,178; Combined = 1,705; Low = 1,458; High = 3,090). The Control treatment had the greatest number of calls detected by the far microphone, followed by the near microphone, and then the middle microphone; the High treatment had greater numbers of pulses detected by both the middle and far microphones than the near; and the High, Low and Combined treatments had the greatest number of pulses detected by the far microphone (Fig. 2). Tricolored bats did not significantly alter their echolocation responses, and no test was significant. However, the lack of observed differences could be due to small sample size (*n* = 8).

## Discussion

We observed species-specific responses to UD emissions and changes in echolocation characteristics that varied by treatment, species and species group, sex, and season. In all cases of significant changes in frequency characteristics, bats echolocated at higher frequencies (*i.e.,* increased Fmin, Fmax, and Fc) during UD emissions. Our results do not suggest that bat species alter echolocation during treatments with UD emissions nearer to their characteristic frequencies. Echolocating bats were predominantly detected by the far microphones, implying bats spent more time on the opposite end of the flight cage from the UD, particularly during UD treatment emissions. Because higher frequencies attenuate more rapidly than lower frequencies, bats located farther from the UD would experience relatively faint UD emissions during the High treatment. This may explain why bats were detected calling more frequently during the High treatment by the middle microphone. In contrast, bats flying at the opposite end of the flight cage during the Low treatment still experienced relatively high intensity UD emissions, which may have influenced their behavior regardless of their echolocation range. Brazilian free-tailed bats, cave myotis, and red bats increased some portion of their echolocation frequencies during the Low and Combined treatments compared to the Control, and evening bats increased a portion of their echolocation frequencies during all three treatments. Similarly, Gillam & Montero (2016) also reported Brazilian free-tailed bats shifting their calls to higher frequencies when exposed to playback signals above their own call. Tressler & Smotherman (2009) noted Brazilian free-tailed bats shifted this echolocation to higher frequencies during playback stimuli of band-limited noise centered at 32.5 kHz.

Cave myotis, a species with typical characteristic and high frequencies of ∼40 and 75 kHz, relatively, (California State Polytechnic University & Humboldt State Bat Lab, 2024) was the only species to increase Fdiff and did so during Low and Combined treatments. The change in Fdiff was mostly attributed to an increase in Fmax and suggests this species increased its bandwidth to discern noise emitted from the UD from echoes returned by the surrounding environment. Short broadband calls are considered advantageous in cluttered environments, as increasing echolocation bandwidth allows for discrimination of prey from background noise (Schnitzler & Kalko, 2001). Additionally, high frequencies attenuate at a faster rate than low frequencies, potentially reducing the time needed for objects to be detected, allowing bats to navigate around clutter in the immediate vicinity (Schnitzler & Kalko, 2001; Gillam et al., 2009). Bats that echolocate using broadband frequencies are more susceptible to spectral overlap and may therefore be more adapted to avoid UD interference than bats that echolocate using narrower bandwidths (Ulanovsky et al., 2004).

Three species changed PD: red bats and evening bats increased PD across all treatments, and Brazilian free-tailed bats decreased PD during Low and Combined treatments. The presence of UD noise may have imitated an increase in acoustic clutter. Species adapted to fly in open air with background clutter (*e.g.*, near edge habitat), such as red bats and evening bats, tend to have relatively intermediate PD (Schnitzler & Kalko, 2001). Increasing their PD during UD treatment may have been the easiest response, which resembles species that are more adapted to foraging in highly cluttered space and have relatively longer duration calls. Brazilian free-tailed bats emit shorter pulses when exposed to acoustic clutter, such as during cave emergences with hundreds to thousands of conspecifics (Schnitzler & Kalko, 2001). The UD treatments may have elicited a similar response from this species to improve their echo reception and reduce potential collisions (Gillam et al., 2010). In contrast, Tressler & Smotherman (2009) reported an increase in PD for Brazilian free-tailed bats when exposed to broadband and band-limited ultrasound in a flight cage. Difference in flight cage configurations may account for the contradictory results. Our flight cage was 60 m × 10 m × 4.4 m with no walls, whereas the cage used by Tressler & Smotherman (2009) was 8 m × 2 m × 3 m cage with sound-absorbing walls.

Bats have evolved traits to adjust their echolocation characteristics in the presence of noise to minimize interference (Gillam & McCracken, 2007; Tressler & Smotherman, 2009). This “jamming avoidance response” has been observed for several species when encountering ultrasound from conspecific and heterospecific bats, and artificial playback noise (Bates, Stamper & Simmons, 2008; Chiu, Xian & Moss, 2009; Gillam & Montero, 2016; Gillam & McCracken, 2007; Habersetzer, 1981; Ibáñez et al., 2004; Necknig & Zahn, 2011; Obrist, 1995; Ratcliffe et al., 2004; Surlykke & Moss, 2000; Ulanovsky et al., 2004). Jamming avoidance response has been shown to elicit microscale shifts in spectral and temporal characteristics of echolocation (Bates, Stamper & Simmons, 2008; Tressler & Smotherman, 2009; Jarvis et al., 2010; Jones, Wohlgemuth & Conner, 2018), but shifts in echolocation by as little as 1 kHz may still be biologically relevant given the intricate nature of sound and bat echolocation.

Alternatively, evidence suggests that bats may instead be exhibiting a Lombard response, which is an involuntarily increase in some echolocation characteristics, such as intensity, duration, and frequency. The Lombard response can improve the signal-to-noise ratio to help bats distinguish their calls from extraneous noise (Amichai, Blumrosen & Yovel, 2015; Cvikel et al., 2015; Gotze et al., 2016; Hage et al., 2013; Hase et al., 2018; Luo, Kothari & Moss, 2017; Tressler & Smotherman, 2009). Pederson et al. (2024) reported that bats evolved a ‘super-fast’ and strong Lombard response to improve sonar tracking and interception of prey in situations with rapidly changing noise levels near conspecifics or calling insects and are uniquely adapted to navigate in such environments.

Although this study was not designed to assess the mechanism for the response, our results are consistent with other studies showing that bats exhibit some flexibility with their echolocation in acoustically cluttered airspace. Whether alterations to certain echolocation characteristics are enough for individuals to navigate in the presence of UDs remains unclear, but they could potentially explain bat flight behavior observed during these treatments. For example, Fritts et al. (2024), who assessed the flight behavior of the same bats during these trials, suggested that Brazilian free-tailed bats fly farther from the UD during the Combined treatment, which correlated with results herein that demonstrated the greatest differences and lowest variances in Fmin, Fc, and PD compared to the Control. Similarly, although all treatments were effective at altering cave myotis flight patterns, the Low and Combined treatments moved this species farther from the UD (Fritts et al., 2024). Acoustically, cave myotis changed only Fmax, Fc, and Fdiff during these two treatments. Evening bats, however, altered flight behavior during all treatments (Fritts et al., 2024) and exhibited flight behavior results consistent with their echolocation results, as all treatments differed from the Control in Fmin, Fc, and PD. Similar to evening bats, all emissions resulted in red bats flying farther from the UD compared to the Control, and PD increased during all of the treatments as well. The species-specific differences could reflect variation in how bats attempt to evade the potential effects of the UD given their echolocation capabilities, behavior, and morphology.

Several factors may contribute to the frequency shifts detected in echolocation between seasons, both during the treatments and compared to the Control. Research suggests that echolocation is a learned behavior (Jones & Ransome, 1993), which could explain differences in echolocation by naïve juvenile bats in the fall season compared to individuals with months of experience echolocating by the following spring season. However, we did not attempt to distinguish juvenile from adults in our study because of the challenges of doing so using the epiphyseal diaphyseal joint outside the summer period. For this reason, because sample sizes of known juveniles and adults were limited for each species, we did not analyze comparisons between adults and juveniles in this study. Seasonal echolocation differences may have contributed to bats altering echolocation based on ambient temperature and seasonal weather variables, which influences bat body temperature and atmospheric attenuation (Jones & Ransome, 1993; Wu et al., 2021). Weather variables having an influence on bat echolocation, as well as attenuation of UD emissions, may together influence UD effectiveness. The differences in echolocation characteristics between seasons by sex that we observed in Brazilian free-tailed bats, red bats, and evening bats could be explained by hormonal shifts that occur throughout the year (particularly during the mating season) that influence echolocation characteristics, as has been documented in *Eptesicus fuscus* in a laboratory setting (Grilliot, Burnett & Mendonça, 2014), Because we only conducted one set of experiments each season, it is also possible that the observed differences were within the range of expected variation.

For red bats, we were able to compare echolocation behavior in the presence of UD emission between males and females within the same season. These results strongly suggest that echolocation varies between sex for this species group, with males echolocating at higher frequencies than females across all initial control and treatment periods for Fmin, Fmax, and Fc. Other bat species, such as evening bats, have been documented to exhibit sexual segregation in foraging habits and habitat selection (Hein, Miller & Castleberry, 2009; Istvanko, Risch & Rolland, 2016), which could indicate that males and females evolved different echolocation frequencies based on their habitats and phenology. However, this seems an unlikely explanation for red bats, as previous research indicates that males and female*s* forage in the same habitat type (Elmore, Miller & Vilella, 2005; Kurta, 2010). The more plausible explanation for echolocation differences between male and female red bats lies with their differences in body size, with female red bats being larger than male red bats (Williams & Findley, 1979). Sexual size dimorphism may be associated with females producing more than one offspring per year, requiring females to fly with and produce nutrients for multiple pups at a single time (Barclay & Harder, 2003; Ammerman, Hice & Schmidly, 2012). Regarding echolocation, body size has shown to be a negative predictor of peak echolocation frequency; that is, as bat body size increases, peak echolocation frequency decreases (Thiagavel, Santana & Ratcliffe, 2017). This is likely due to smaller bats having smaller larynges, which constrain them to producing higher frequencies (Thiagavel, Santana & Ratcliffe, 2017).

The effectiveness of UDs at reducing bat fatalities at wind turbines is variable, with mixed results detected among species at a single wind energy facility and within species at different wind energy facilities (Arnett et al., 2013; Romano et al., 2019; Weaver et al., 2020; Clerc et al., 2025). Several UD technologies and placement locations on wind turbines have been investigated, which may explain some of this variation. It is also possible that the ultrasound emitted lacked the intensity (decibels) necessary to extend far enough to deter bats before they reach the rotor-swept area. To date, all UD technologies have been installed on the wind turbine tower or nacelle. Because ultrasound attenuates relatively rapidly, a 30 kHz signal decreases approximately 70 dB within about 50 m from the source (Weaver et al., 2020). The sound pressure intensity necessary to deter bats is unknown, but this rate of attenuation is likely to reduce the effectiveness of UDs. Bat may also become habituated to the UD signal. With one exception, UD technologies have emitted a constant broadband signal, to which bats may become accustomed. Romano et al. (2019) experimented with a pulsed signal, but water collected in the nozzles of the air-jet system and distorted the ultrasonic signal. Habituation in bats is not well studied, but there is some evidence that bats do habituate to anthropogenic noise and playbacks (Luo et al., 2014; Dixon et al., 2019). Finally, bats may rely more on vision than echolocation when near wind turbines. Jonasson et al. (2025) demonstrated that hoary bats and silver-haired bats were 74% and 97% more likely to fly toward an illuminated section of a wind turbine blade than the alternative dark open flyway.

Our results showed that the high frequency treatment had no effect on Brazilian free-tailed bats or cave myotis, altered only one characteristic (PD) for red bats, and was no different than the other two treatments for evening bats. Given the lack of effectiveness of the high frequency treatment and the reduced ability to broadcast frequencies in this range (36–50 kHz) the full length of wind turbine blades, it may not be necessary for UDs to include frequencies above 36 kHz. Eliminating higher frequencies would allow UDs to focus on the lower frequency bandwidth which can extend beyond the length of most wind turbine blades.

## Conclusions

This study and Fritts et al. (2024) demonstrated that bats change their acoustic and flight behavior, respectively, when exposed to UD signals intended to deter bats from wind turbines. Regardless of the mechanism used (*i.e.,* either jamming avoidance response or Lombard response), the ability of a bat to navigate in the presence of UDs may reflect the variability in species-specific fatalities observed during experimental studies of UDs on wind turbines. Two of the species studied here, red bats and Brazilian free-tailed bats, responded differently to the UD treatments in the flight cage. Weaver et al. (2020) reported a significant reduction in Brazilian free-tailed bat fatality with UDs, whereas Clerc et al. (2025) reported a significant increase in eastern red bat fatalities with UDs; both studies used the same UD technology, which was also used in this study. Given this variability in fatality reductions using the existing UD technology (*i.e.,* constant broadband signal emitted from the tower or nacelle), alternative placements (*e.g.*, wind turbine blades) or emissions (*e.g.*, randomized pulses) are potential areas of focus to improve UD technology. Based on our results, for example, UDs could concentrate their emissions on lower ultrasonic frequencies (*i.e.,* approximately less than 36 kHz) to optimize the effectiveness of the technology. In addition, an alternate minimization strategy such as curtailment may be needed in conjunction with UDs to minimize bat fatalities from wind turbines (*e.g.*, Good et al., 2022).

##  Supplemental Information

10.7717/peerj.21231/supp-1 Supplemental Information 1 TABR raw data

10.7717/peerj.21231/supp-2 Supplemental Information 2 MYVE raw data

10.7717/peerj.21231/supp-3 Supplemental Information 3 LABO raw data

10.7717/peerj.21231/supp-4 Supplemental Information 4 NYHU raw data

10.7717/peerj.21231/supp-5 Supplemental Information 5 PESU raw data

## Additional Information and Declarations

### Data Availability

The following information was supplied regarding data availability: The raw data are available in Supplemental Files.
